# The mutational spectrum in whole exon of *p53* in oral squamous cell carcinoma and its clinical implications

**DOI:** 10.1038/s41598-022-25744-8

**Published:** 2022-12-15

**Authors:** Toshiki Hyodo, Nobuyuki Kuribayashi, Chonji Fukumoto, Yuske Komiyama, Ryo Shiraishi, Ryouta Kamimura, Yuta Sawatani, Erika Yaguchi, Tomonori Hasegawa, Sayaka Izumi, Takahiro Wakui, Koh-ichi Nakashiro, Daisuke Uchida, Hitoshi Kawamata

**Affiliations:** 1 Department of Oral and Maxillofacial Surgery, Dokkyo Medical University School of Medicine, 880 Kita-Kobayashi, Mibu, Shimotsuga, Tochigi 321-0293 Japan; 2 Department of Oral and Maxillofacial Surgery, Ehime University Graduate School of Medicine, 454 Shitsukawa, Toon, Ehime 791-0295 Japan

**Keywords:** Cancer genetics, Cancer genomics, Oral cancer

## Abstract

Mutations in *p53* are common in human oral squamous cell carcinoma (OSCC). However, in previous analyses, only detection of mutant p53 protein using immunohistochemistry or mutations in some exons have been examined. Full length mutant p53 protein in many cases shows a loss of tumor suppressor function, but in some cases possibly shows a gain of oncogenic function. In this study, we investigate relationships of outcomes with the mutational spectrum of *p53* (missense and truncation mutations) in whole exon in OSCC. Specimens from biopsy or surgery (67 cases) were evaluated using next-generation sequencing for *p53*, and other oncogenic driver genes. The data were compared with overall survival (OS) and disease-free survival (DFS) using univariate and multivariate analyses. *p53* mutations were detected in 54 patients (80.6%), 33 missense mutations and 24 truncation mutations. *p53* mutations were common in the DNA-binding domain (43/52) and many were missense mutations (31/43). Mutations in other regions were mostly *p53* truncation mutations. We detected some mutations in 6 oncogenic driver genes on 67 OSCC, 25 in *NOTCH1*, 14 in *CDKN2A*, 5 in *PIK3CA*, 3 in *FBXW7*, 3 in *HRAS*, and 1 in *BRAF*. However, there was no associations of the *p53* mutational spectrum with mutations of oncogenic driver genes in OSCC. A comparison of cases with *p53* mutations (missense or truncation) with wild-type *p53* cases showed a significant difference in lymph node metastasis. DFS was significantly poorer in cases with *p53* truncation mutations. Cases with *p53* truncation mutations increased malignancy. In contrast, significant differences were not found between cases with *p53* missense mutations and other mutations. The *p53* missense mutation cases might include cases with mostly similar function to that of the wild-type, cases with loss of function, and cases with various degrees of gain of oncogenic function.

## Introduction

Oral cancer is a general term for malignant epithelial tumors developing in the buccal mucosa, maxillary gingiva, mandibular gingiva, hard palate, tongue, and oral floor^1^. The Union for International Cancer Control (UICC) also includes malignant epithelial tumors developing in the lips as oral cancer^2^. Oral cancer is composed of more than 90% of squamous cell carcinoma and other malignant epithelial tumors including salivary gland cancers^1^. There are no available worldwide statistics for oral cancer alone, but it is estimated that 370,000 patients develop oropharyngeal cancer yearly and more than 170,000 die from this disease^3^. Oral squamous cell carcinoma (OSCC) is highly locally invasive and lymph node metastasis is common in advanced cases. The incidence of distant metastasis is relatively low, but the outcome is poor if lung metastasis occurs^4,5^. OSCC treatment involves multidisciplinary therapy with surgery, chemotherapy, and radiotherapy, and molecularly targeted drugs and immune checkpoint inhibitors have recently become available^6–8^. The malignancy grade of OSCC can be determined histopathologically or genetically, but neither of these methods is definitive^9^. We have evaluated malignancy based on the origin of OSCC and we have found higher malignancy and poorer outcomes for OSCC that developed from bipotential stem cells in salivary gland^10^ or undifferentiated stem cells form bone marrow^11^.

*p53* is a closely investigated tumor suppressor gene that has been referred to as ‘the guardian of the genome’. Mutations in *p53* are common in most human cancers^12–14^. However, in the early studies of *p53* abnormalities, only detection of mutant p53 protein with a long half-life using immunohistochemistry (IHC) or mutations in some exons have been examined^15,16^. Truncation mutations such as nonsense mutation, frame-shift, and splice-site variants cannot be detected using IHC and categorized in IHC-negative case (wild-type). Moreover, mutations other than hot spots are frequently overlooked by direct sequencing or single strand conformation polymorphism analysis. Recently, the entire exome of tumor suppressor genes and oncogenes, including *p53*, were analyzed in many tumors at TCGA^17^, but there is no analysis in the Japanese population. In addition, there was no analysis of the pattern of mutations in the *p53* gene by region, nor was there any analysis or discussion of the functional diversity of *p53* missense mutations. Furthermore, although *p53* mutation and abnormal protein accumulation can be detected in these analyses, the kind of functional abnormality is unclear. In our previous studies on *p53* abnormality in oral cancer^18–26^, full length mutant p53 protein was produced in many cases with *p53* mutations, indicating a lack of rationality with regard to functional loss of the tumor suppressor gene. In an oral cancer cell line with a *p53* missense mutation, we have shown that transcriptional activity of a *p53* target gene differs depending on the missense mutation site, suggesting that a full-length mutant p53 protein can have oncogenic effects^27^.

Kotler et al. recently reported a construction of systematic library for analysis of functional abnormalities of mutations in the *p53* DNA-binding domain (DBD), in a study of the association of the mutation site and pattern with the malignancy grade^28^. This suggests the importance of dividing *p53* abnormalities into missense and truncation mutations, and dividing wild-type cases into those with and without oncogenic human papillomavirus (HPV) infection. In addition, it may be important to identify the type of functional abnormality, as loss of function or gain of function (several types); i.e., diagnosis of the mutational spectrum of *p53*.

In this study, we investigated the relationships between the mutation sites and patterns [missense mutations or truncation mutations (nonsense mutation, frame-shift variant, splice-site variant, or in-frame deletion)] in whole *p53* exons in OSCC tissue, and IHC p16-positivity (as a surrogate marker for oncogenic HPV infection and integration) and activating mutations of OSCC oncogenic driver genes (*BRAF, CDKN2A, FBXW7, HRAS, NOTCH1, PIK3CA*). Then, we attempted to clarify the associations between theses gene abnormalities and the biological and clinical malignancy of OSCC, based on TNM classification, Yamamoto–Kohama (Y–K) mode of invasion^29,30^, degree of differentiation, overall survival (OS), and disease-free survival (DFS).

## Patients and methods

### Patients

Sixty-seven patients with primary OSCC treated at Dokkyo Medical University Hospital Oral and Maxillofacial Surgery and Ehime University Hospital Dentistry, Oral Surgery and Orthodontics between 2015 and 2020 were subjected to the study (Table 1). All tumors examined are located in the oral cavity but not in the oropharyngeal region. Observation period of the patients was from the sampling date or day of surgery to the final hospital visit. The study was approved by Dokkyo Medical University Hospital Ethics Committee (R-20-19-J) and Ehime University Hospital Ethics Committee (No. 2008016) or by Ehime University Human Genome/Gene Analysis Research Ethics Committee (R2-16). The study was opt-out and no patient wished to be excluded. Informed consent was also obtained from all patients.

**Table 1:** Characteristics of the patients and the *p53* mutational spectrum.

	n = 67 (cases)	Missense mutation	Truncation mutation	Wild-type/*p16* +	Wild-type/*p16-*
**Age**					
< 65 years	25	9	14	2	0
≥ 65 years	42	22	9	4	7
**Gender**					
Male	39	18	15	5	1
Female	28	13	8	1	6
**Smoking**					
Smoking	34	16	12	5	1
Non smoking	33	15	11	1	6
**Drinking**					
Drinking	30	14	10	4	2
Non drinking	37	17	13	2	5
**UICC pathological T category**					
pT1	8	5	1	1	1
pT2	21	9	8	1	3
pT3	15	6	7	2	0
pT4a	17	9	5	1	2
pT4b	6	2	2	1	1
**UICC pathological N category**					
pN0	39	18	9	5	7
pN1	13	7	5	1	0
pN2a	5	3	2	0	0
pN2b	7	1	6	0	0
pN2c	2	1	1	0	0
pN3a	0	0	0	0	0
pN3b	1	1	0	0	0
**UICC pathological stage**					
Stage I	8	5	1	1	1
Stage II	13	5	5	0	3
Stage III	18	10	5	3	0
Stage IVa	22	9	10	1	2
Stage IVb	6	2	2	1	1
**Tumor cell differentiation**					
Well	29	12	9	4	4
Moderate	19	10	6	1	2
Poor	19	9	8	1	1
**Y–K mode of invasion**					
1	9	5	1	1	2
2	14	6	3	2	3
3	28	15	12	0	1
4C	15	4	7	3	1
4D	1	1	0	0	0

### Histopathological examination

Histological samples were fixed with formalin and embedded in paraffin. Sections of 4-μm were stained with hematoxylin and eosin, and a pathological diagnosis was then made by experienced pathologists. Tumor cell differentiation (World Health Organization: WHO classification) and Y–K mode of invasion were evaluated by a co-author (HK).

### Immunohistochemical staining

Using specimens resected in biopsy or surgery, immunohistochemical staining was performed for p53 and p16. Resected tissue was immediately fixed with 10% neutral-buffered formalin solution and paraffin-embedded to prepare 4-μm thin sections. The sections were deparaffinized with xylene and serially rehydrated with ethanol. Antigen was activated by microwave at 95 °C for 10 min (pH 6.0 citrate buffer solution), washed with phosphate-buffered saline (PBS), and then treated with 0.3% hydrogen peroxide in methanol for 20 min for inhibition of endogenous peroxidase; a 30-min total blocking time. X0909 Protein Block Serum-Free (Dako, Glostrup, Denmark) was used for blocking. Incubations with mouse anti-human p53 monoclonal antibody (Clone DO-7, 1:50 dilution, Dako) and mouse anti-human p16 monoclonal antibody (Clone G175-405, 1:200 dilution, BD Pharmingen, San Diego, CA) as primary antibodies were performed for 60 min. Thereafter, the procedure followed the polymer-immune complex method using Envision (K4001, Dako). In p53 and p16 immunostaining, a stain coinciding with a tumor cell nucleus was regarded as positive^24,31^. Samples were evaluated by TH (Toshiki Hyodo) and HK using measurements in ≥ 5 visual fields at 200 × magnification: cases with no positive findings were regarded as negative, and those with a positive finding in one or more visual fields were regarded as positive.

### DNA extraction

From each patient with OSCC, genomic DNA (gDNA) was extracted from an approximately 25-mg specimen from the tumor center in the biopsied or surgically excised material using a QIAamp Fast DNA Tissue Kit (Qiagen, Hilden, Germany)^7,32^. About 100 ng/7.5 µl of gDNA was used for the next-generation sequencing (NGS). Histological analysis was performed on the remaining specimen to confirm the presence of active tumor cells.

### Procedure for NGS

A library was prepared using an AmpliSeq for Illumina Custom DNA Panel (Illumina, San Diego, CA, USA). We originally customized this panel for OSCC to detect the gene abnormalities of *p53* full coding sequence (CDS), *BRAF* (exon 15), *CDKN2A* (full CDS), *FBXW7* (full CDS), *HRAS* (exons 2 and 3), *NOTCH1* (full CDS), and *PIK3CA* (exons 10 and 21)^7^. The concentration of the library was adjusted for each sample. After mixing of each sample, the library was sequenced by a Next-Generation Sequencer (MiSeq, Illumina) (300 cycles) with a MiSeq Reagent Kits v2 (Illumina). Altered Variant Frequency was set at a cut-off of ≥ 5% and Read Depth at ≥ 500 reads as NGS mutation criteria.

Nucleotide sequence data reported are available in the DDBJ Sequenced Read Archive under the accession numbers DRA014726.

### Statistical analysis

Cases with *p53* mutation and wild-type cases were compared by Chi-square test, with *p* < 0. 05 regarded as significant. In univariate and multivariate analyses, age, gender, smoking, drinking, UICC TNM classification, Y–K mode of invasion, *p53* mutational spectrum (missense mutation, truncation mutation, wild-type/*p16* status) were included as potential risk factors. Hazard ratios (HR) were calculated in a Cox proportional hazard model, again with *p* < 0. 05 regarded as significant. A two-sided 95% confidence interval was also calculated. The 5-year OS and DFS were evaluated by Kaplan–Meier analysis and Log-rank test for each type of *p53* mutation. IBM SPSS ver. 27. 0 (IBM SPSS, Inc., Tokyo, Japan) was used in all statistical analyses.

### Ethics declarations

This study was conducted in accordance with the Declaration of Helsinki and approved by Dokkyo Medical University Hospital Ethics Committee (R-20-19-J) and Ehime University Hospital Ethics Committee (No. 2008016) or by Ehime University Human Genome/Gene Analysis Research Ethics Committee (R2-16). The study was opt-out and no patient wished to be excluded. Informed consent was also obtained from all patients.

## Results

### Characteristics of the patients and the *p53* mutational spectrum

Sixty-seven patients with primary OSCC including 39 males (58.2%) and 28 females (41.8%) who underwent radical tumor resection were enrolled in this study (Table 1). The mean age was 70.9 (30–98) years old, with 25 patients aged < 65 and 42 aged ≥ 65 years old. There were 34 smokers and 33 non-smokers, and 30 patients drank alcohol and 37 were non-drinkers. The pathological T category in the UICC TNM classification was pT1 in 8, pT2 in 21, pT3 in 15, pT4a in 17, and pT4b in 6 cases. The pathological N category in the UICC TNM classification was pN0 in 39, pN1 in 13, pN2a in 5, pN2b in 7, pN2c in 2, pN3a in 0, and pN3 b in 1 case. All the patients did not show any distant metastasis at the primary operation. The pathological UICC staging was Stage I in 8, Stage II in 13, Stage III in 18, Stage IVa in 22, and Stage IVb in 6. On tumor cell differentiation, well-differentiated case was 29, moderately differentiated case was 19, and poorly differentiated case was 19. On the Y-K mode of invasion, Y-K-1 case was 9, Y-K-2 case was 14, Y-K-3 case was 28, Y-K-4C case was 15, and Y-K-4D case was 1.

### *p53* mutational spectrum

**Figure 1:**
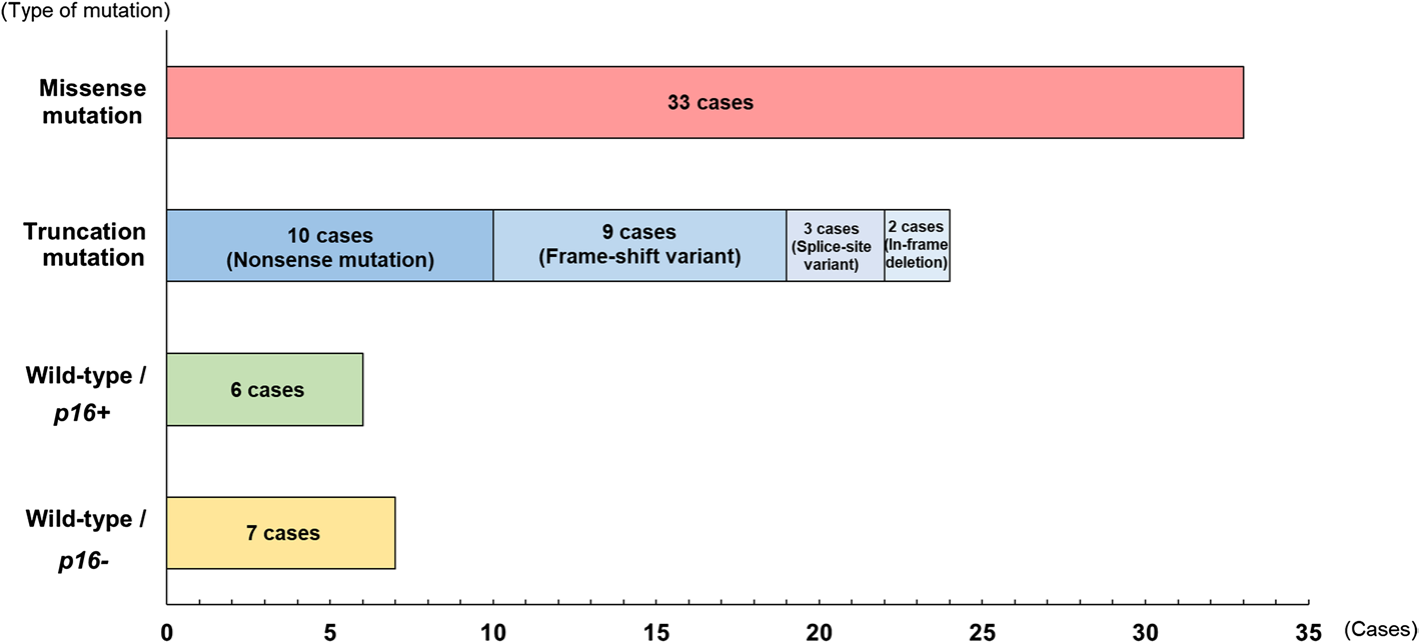
*p53* mutational spectrum. *p53* mutation was found in 54 of 67 patients (80.6%), and a total of 57 mutations were detected, including 3 cases with double mutations. These included 33 missense mutations and 24 truncation mutations (nonsense mutation 10, frame-shift variant 9, splice-site variant 3, in-frame deletion 2). There were 6 *p16-*positive and 7 *p16*-negative wild-type cases.

**Figure 2:**
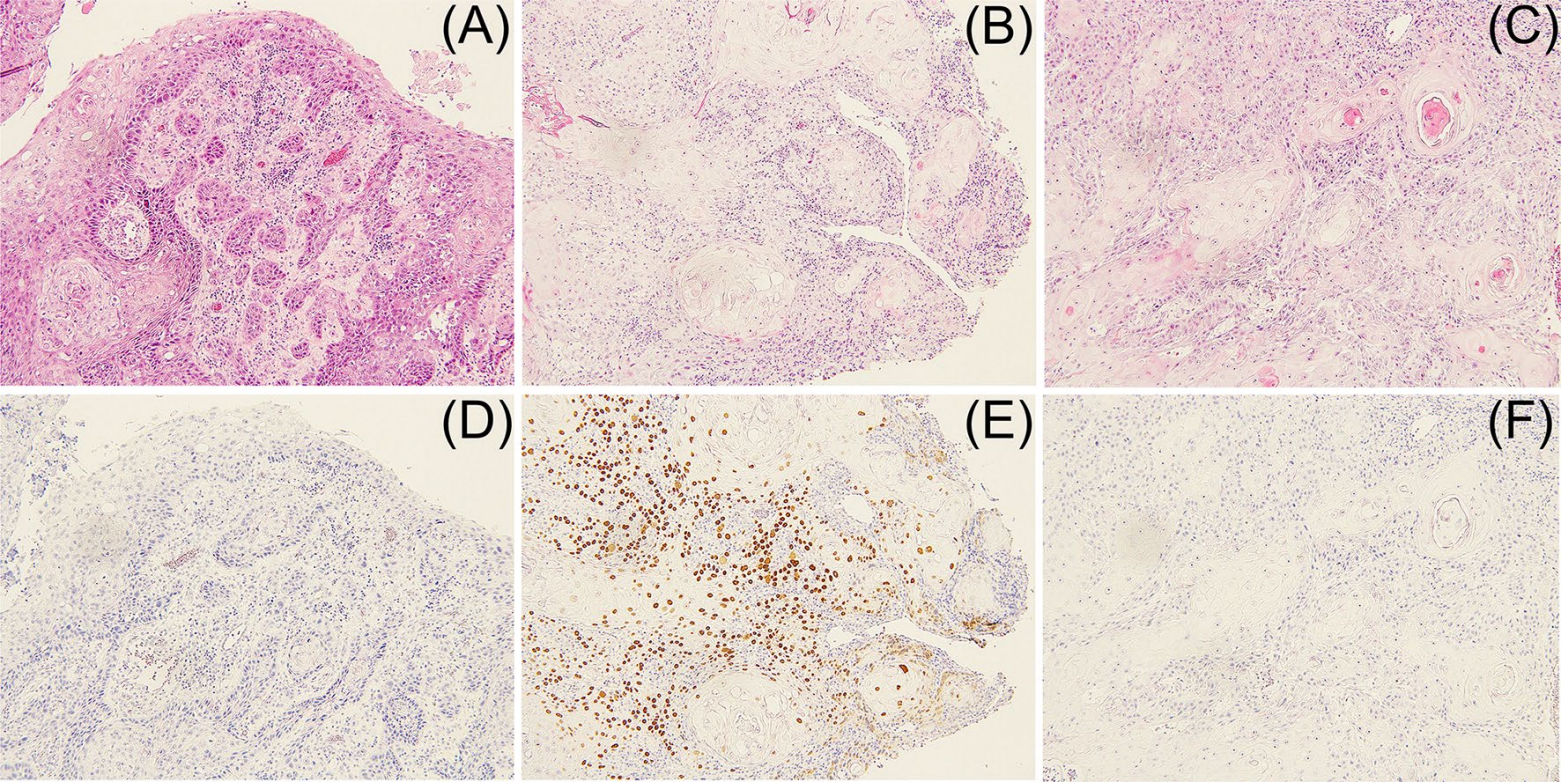
Immunohistological evaluation of p53 in oral squamous cell carcinoma (OSCC) cases. No abnormal accumulation of p53 protein in the nucleus was observed in cases in which no mutation was found by next-generation sequencing (NGS) (**A**,**D**). In cases with a mutation detected by NGS, abnormal nuclear accumulation of p53 protein was found immunohistologically (**B**,**E**). In cases with a truncation mutation detected by NGS, the absence of abnormal nuclear accumulation of p53 protein was shown immunohistologically (**C**,**F**).

*p53* mutation was found in 54 of 67 patients (80.6%), and a total of 57 mutations were detected, including 3 cases with double mutations (Fig. 1). These included 33 missense mutations and 24 truncation mutations (nonsense mutation 10, frame-shift variant 9, splice-site variant 3, in-frame deletion 2). In the wild-type cases, immunohistology showed that 6 and 7 patients were p16*-*positive (oncogenic HPV was infected and integrated) and-negative (oncogenic HPV was not infected), respectively. For verification of the *p53* mutations, the immunohistological and NGS results were evaluated in combination. No abnormal accumulation of p53 protein in the nucleus was observed immunohistologically in cases in which NGS indicated no mutation (Fig. 2A,D). In contrast, cases in which a mutation was identified by NGS had abnormal nuclear accumulation of p53 protein immunohistologically (Fig. 2B,E). In cases in which mutation was unclear on NGS despite observation of p53 protein accumulation in the nucleus, the read depth decreased from 500 to 200 and the presence of a mutation was confirmed by reinvestigation. In cases with a *p53* truncation mutation detected by NGS, the absence of abnormal p53 protein accumulation in the nucleus was confirmed immunohistologically (Fig. 2C,F).

### *p53* mutational landscape in OSCC

**Figure 3:**
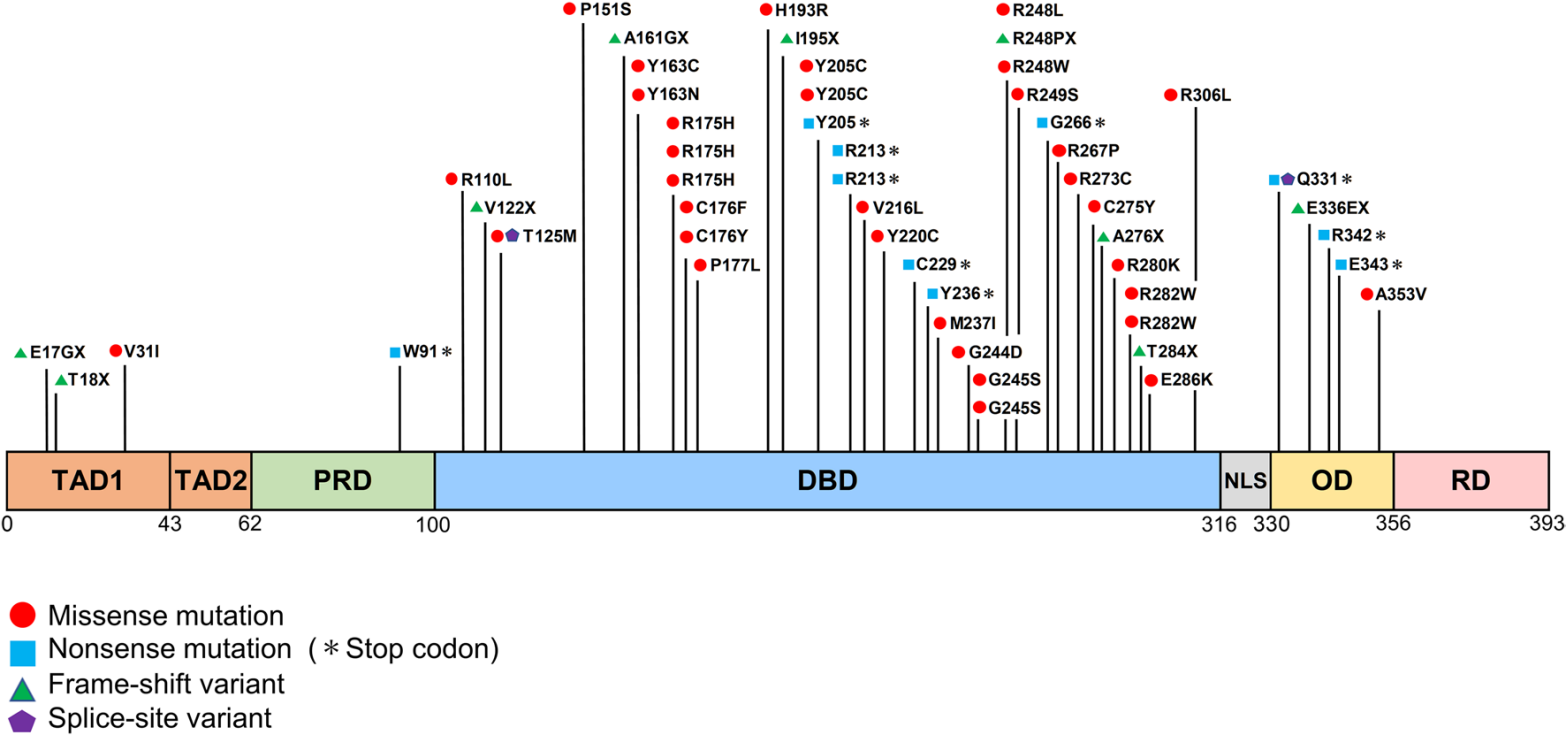
*p53* mutational landscape in oral squamous cell carcinoma (OSCC). The distribution of *p53* mutations was diverse, but many were present in the DNA-binding domain (DBD) (43/52) and these were mainly missense mutations (31/43). A truncation mutation of the DBD was found in 13/43 cases. In other regions (transactivation domain: TAD, proline-rich domain: PRD, oligomerization domain: OD) most mutations were truncation mutations (7/9), with missense mutation found in only 2 cases.

The *p53* mutational spectrum in OSCC showed a diverse distribution of mutations (Fig. 3). However, mutations were common in the DBD (43/52) and many of these were missense mutations (31/43). Some truncation mutations were also present in the DBD (13/43). Mutations in other regions, such as the transactivation domain (TAD), proline-rich domain (PRD), and oligomerization domain (OD) (7/9), were mostly truncation mutations, with missense mutations found in only 2 cases.

### Relationship between the *p53* mutational spectrum and mutations of oncogenic driver genes in OSCC

Relationships between the *p53* mutational spectrum in OSCC and mutations of oncogenic driver genes are shown in Table 2. The term "oncogenic driver genes" in this manuscript includes activation of oncogenes (*BRAF, HRAS, PIK3CA*) and inactivation of tumor suppressor genes (*p53, CDKN2A, FBXW7, NOTCH1*). *BRAF* was mutated in only 1 case (3.2%) with a *p53* missense mutation; *CDKN2A* was mutated in 6 cases (19.4%) with a *p53* missense mutation and 8 (34.8%) with a *p53* truncation mutation; *FBXW7* was mutated in 1 case (3.2%) with a *p53* missense mutation and 2 (8.7%) with a *p53* truncation mutation; *HRAS* was mutated in 1 case (3.2%) with a *p53* missense mutation and 2 (15.4%) with wild-type *p53*; *NOTCH1* was mutated in 11 cases (35.5%) with a *p53* missense mutation, 6 (26.1%) with a *p53* truncation mutation, and 8 (61.5%) with wild-type *p53*; and *PIK3CA* was mutated in 2 cases (6.5%) with a *p53* missense mutation, 2 (8.7%) with a *p53* truncation mutation, and 1 case (7.7%) with wild-type *p53*. Regarding double mutations, the case with *p53* missense and truncation (splice-site) mutations was defined as having a *p53* truncation mutation; that with *p53* truncation (nonsense and splice-site) mutations as having a *p53* truncation (nonsense) mutation; and that with two *p53* missense mutations as having a *p53* missense mutation.

**Table 2:** Relationship between the *p53* mutational spectrum and mutations of oncogenic driver genes in OSCC.

*p53* mutational spectrum	*BRAF*	*CDKN2A*	*FBXW7*	*HRAS*	*NOTCH1*	*PIK3CA*
**Missense mutation**	1/31 (3.2%)	6/31 (19.4%)	1/31 (3.2%)	1/31 (3.2%)	11/31 (35.5%)	2/31 (6.5%)
**Truncation mutation**	0/23 (0.0%)	8/23 (34.8%)	2/23 (8.7%)	0/23 (0.0%)	6/23 (26.1%)	2/23 (8.7%)
Nonsense mutation	0/10 (0.0%)	3/10 (12.5%)	0/10 (0.0%)	0/10 (0.0%)	3/10 (30.0%)	0/10 (0.0%)
Frame-shift variant	0/9 (0.0%)	3/9 (33.3%)	1/9 (11.1%)	0/9 (0.0%)	2/9 (22.2%)	2/9 (22.2%)
Splice-site variant	0/2 (0.0%)	2/2 (100.0%)	1/2 (50.0%)	0/2 (0.0%)	1/2 (50.0%)	0/2 (0.0%)
In-frame deletion	0/2 (0.0%)	0/2 (0.0%)	0/2 (0.0%)	0/2 (0.0%)	0/2 (0.0%)	0/2 (0.0%)
**Wild-type**	0/13 (0.0%)	0/13 (0.0%)	0/13 (0.0%)	2/13 (15.4%)	8/13 (61.5%)	1/13 (7.7%)
*p16* +	0/6 (0.0%)	0/6 (0.0%)	0/6 (0.0%)	0/6 (0.0%)	4/6 (66.7%)	0/6 (0.0%)
*p16-*	0/7 (0.0%)	0/7 (0.0%)	0/7 (0.0%)	2/7 (28.6%)	4/7 (57.1%)	1/7 (14.3%)

*Counting of double mutations: Missense + Truncation (Splice-site variant) → Truncation (Splice-site variant); Truncation (Nonsense) + Truncation (Splice-site variant) → Truncation (Nonsense); Missense + Missense → Missense.

### Comparison of mutation rates of oncogenic driver genes in OSCC between this study and a previous study

A comparison of mutations of oncogenic driver genes in OSCC between this study (Japanese population only) and a previous study (Mixed races in USA)^17^ is shown in Table 3. In the current study, *p53* mutation was found in 54 cases (80.6% vs. 72.0%), and there were 25 cases with *NOTCH1* mutation (37.0% vs. 19.0%), 14 with *CDKN2A* mutation (20.9% vs. 22.0%), 5 with *PIK3CA* mutation (7.5% vs. 21.0%), 3 with *FBXW7* mutation (4.5% vs. 2.9%), 3 with *HRAS* mutation (4.5% vs. no data), and 1 with *BRAF* mutation (1.5% vs. no data).

**Table 3:** Comparison of mutation rates of oncogenic driver genes in OSCC between this study and previous study.

Driver gene	Number of cases	Rate (%)	Frequency of mutation in data base^17^
*p53*	54/67	80.6	72.0%
*NOTCH1*	25/67	37.0	19.0%
*CDKN2A*	14/67	20.9	22.0%
*PIK3CA*	5/67	7.5	21.0%
*FBXW7*	3/67	4.5	2.9%
*HRAS*	3/67	4.5	No data
*BRAF*	1/67	1.5	No data

*Counting of double mutations: Missense + Truncation (Splice-site variant) → Truncation (Splice-site variant); Truncation (Nonsense) + Truncation (Splice-site variant) → Truncation (Nonsense); Missense + Missense → Missense.

### Characteristics of the patients and the *p53* mutational spectrum (statistical analysis)

There were no significant differences in the clinical and biological characteristics among cases with a *p53* missense mutation, *p53* truncation mutation, wild-type *p53* and *p16-*positive, and wild-type *p53* and *p16-*negative status (data not shown). However, a comparison of cases with *p53* mutations (missense or truncation) with all wild-type *p53* cases showed a significant difference in pN (*p* = 0.005) and Y-K mode of invasion (*p* = 0.021) by Chi-square test (Table 4). Risk factors for OS and DFS were investigated based on clinical and biological characteristics using binominal logistic regression univariate and multivariate analyses (Tables 5, 6). pN was a significant poor prognostic factor for OS in both analyses (univariate: *p* = 0.002, multivariate: *p* = 0.005) as well as for DFS in both analyses (univariate: *p* = 0.005, multivariate: *p* = 0.026). Y–K mode of invasion was associated with DFS in univariate analysis (*p* = 0.042). DFS also differed significantly in cases with truncation mutations compared with all other cases (missense mutations and wild-type *p16-*positive or negative) in univariate analysis (*p* = 0.050).

**Table 4:** Characteristics of the patients and *p53* mutational spectrum (Statistical analysis).

n = 67 (cases)	Missense + Truncation	Wild-type/*p16* + *, p16-*	*p* value
**Age**			
< 65 years	23	2	
≥ 65 years	31	11	*p* = 0.069
**Gender**			
Male	33	6	
Female	21	7	*p* = 0.326
**Smoking**			
Smoking	28	6	
Non smoking	26	7	*p* = 0.712
**Drinking**			
Drinking	24	6	
Non drinking	30	7	*p* = 0.911
**UICC pathological T category**			
pT1	23	6	
pT2			
pT3	31	7	
pT4a			
pT4b			*p* = 0.816
**Pathological lymph node metastasis**			
Negative (pN0)	27	12	
Positive (pN1, pN2a, pN2b, pN2c, pN3a, pN3b)	27	1	p = 0.005
**UICC pathological stage**			
Stage I	16	5	
Stage II			
Stage III	38	8	
Stage IVa			
Stage IVb			*p* = 0.538
**Y–K mode of invasion**			
1	15	8	
2			
3	39	5	
4C			
4D			p = 0.021
			Chi-square test

**Table 5:**
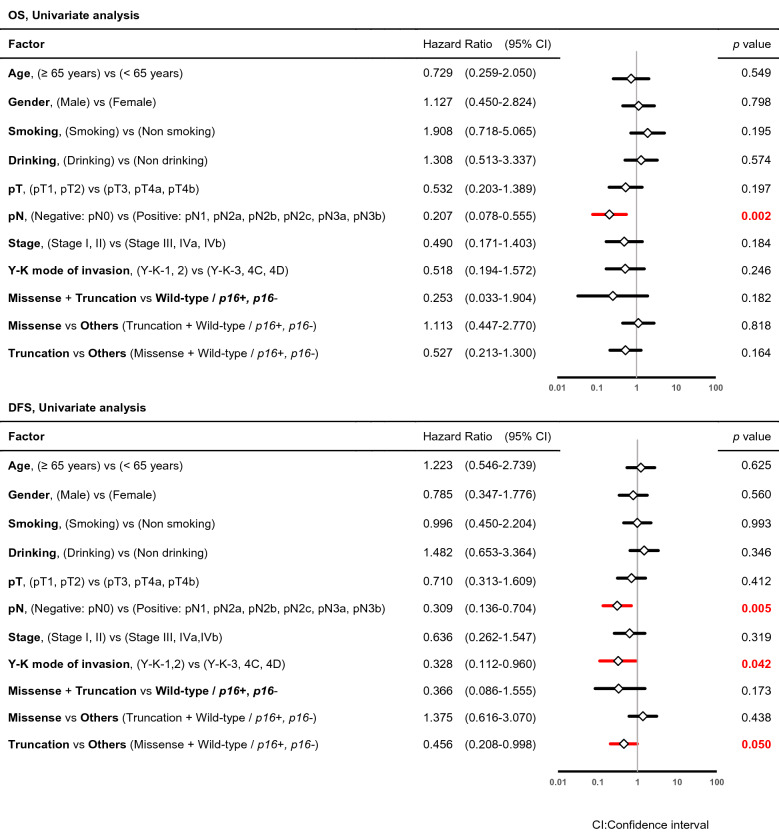
Characteristics of patients and the *p53* mutational spectrum as risk factors for OS and DFS (univariate analysis).

**Table 6:**
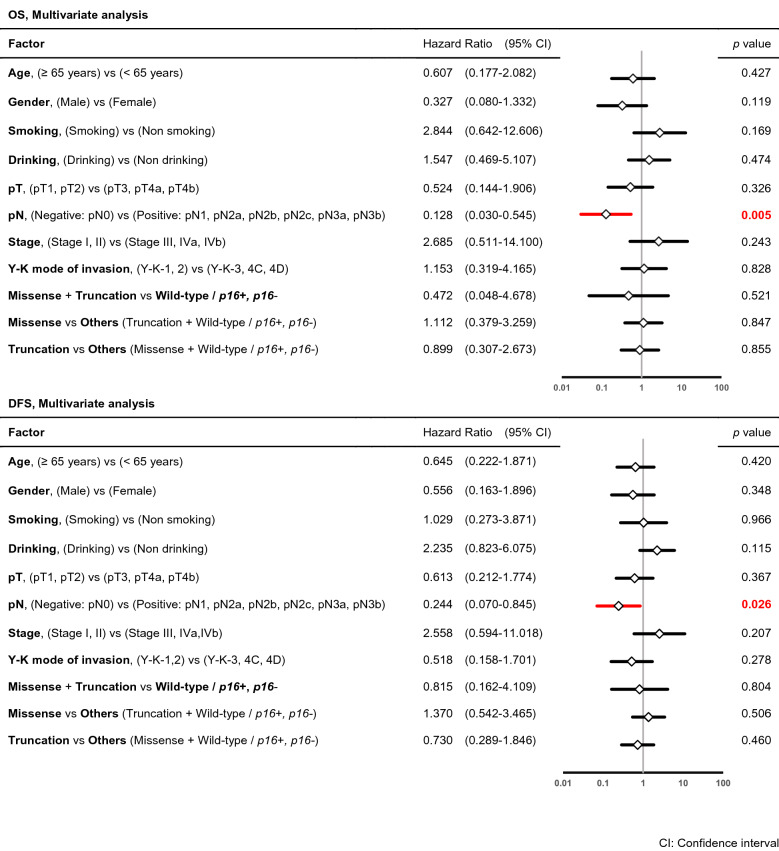
Characteristics of patients and the *p53* mutational spectrum as risk factors for OS and DFS (multivariate analysis).

### Cumulative OS and DFS in OSCC based on the *p53* mutational spectrum

**Figure 4:**
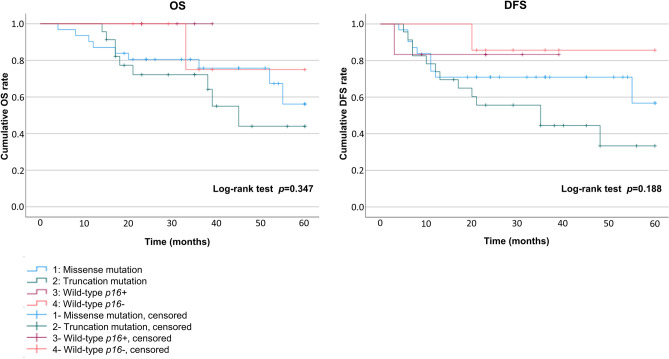
Overall survival (OS) and disease-free survival (DFS) in patients with oral squamous cell carcinoma (OSCC). OS and DFS in patients with OSCC with *p53* missense mutations, *p53* truncation mutations, wild-type *p16* + and wild-type *p16−*. OS and DFS were compared among the four groups using the Kaplan–Meier method with a log-rank test. There were no significant differences in OS (*p* = 0.347) or DFS (*p* = 0.188) among the four groups.

**Figure 5:**
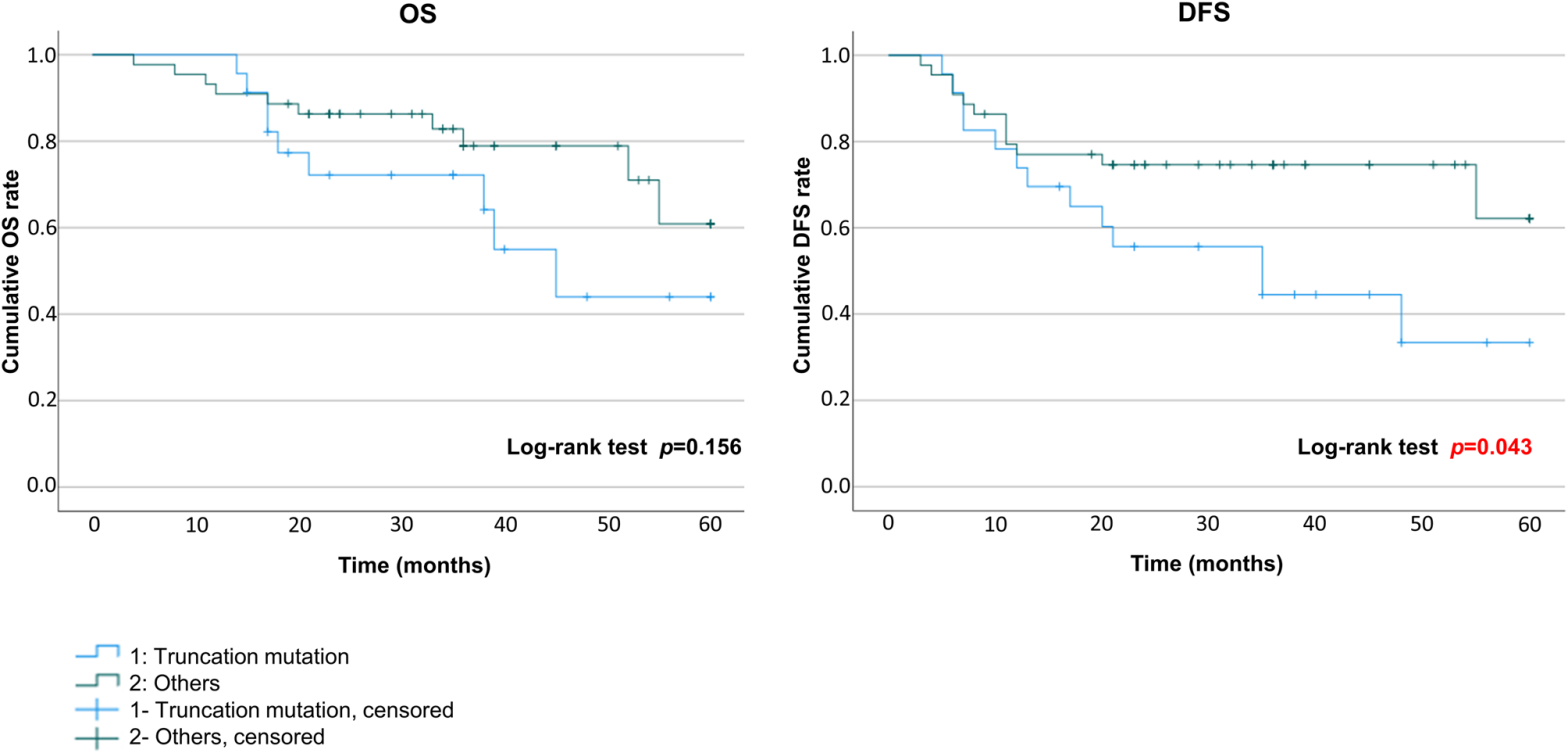
Overall survival (OS) and disease-free survival (DFS) in patients with oral squamous cell carcinoma (OSCC) with *p53* truncation and non-truncation mutations. There was no significant difference in OS (*p* = 0.156) between the groups, but DFS was significantly poorer in patients with truncation mutations (*p* = 0.043).

Patients with OSCC with each type in the *p53* mutational spectrum (missense mutation, truncation mutation, wild-type *p16-*positive, and wild-type *p16-*negative) were evaluated using the Kaplan–Meier method with a log-rank test, but no significant difference in OS (*p* = 0.347) or DFS (*p* = 0.188) was found among the four groups (Fig. 4). However, DFS (*p* = 0.043) was significantly poorer in cases with truncation mutations compared to the other three groups, but OS (*p* = 0.156) did not show this difference (Fig. 5). There was no significant difference in OS or DFS in cases with missense mutations compared with the other three groups (data not shown).

## Discussion

In this study, *p53* mutation was found in 54 of 67 patients (80.6%), and a total of 57 mutations were detected, including 3 cases with double mutation. The *p53* mutation site was diverse, but mostly found in the DBD, and many mutations in the DBD were missense mutations. These findings for OSCC are similar to those in previous reports for other tumors^33,34^. Olivier et al. found occasional missense mutations throughout the coding region, but 97% of these mutations were detected in the exon coding for the DBD^35^. Missense mutation-induced amino acid substitution in the DBD changes DNA binding capacity and leads to a loss or change in transcriptional activation^36^. In the DBD, 6 major hotspots have been identified at codons 175, 245, 248, 249, 273, and 282^37,38^. In the current study, mutations were detected at all of these hotspots, codons 175 (3 cases), 245 (1 case), 248 (3 cases), 249 (1 case), 273 (1 case), and 282 (2 cases), respectively. Surprisingly, in regions other than the DBD, truncation mutations were found in many cases, but only 2 missense mutations. These 2 missense mutations were likely to have been single nucleotide polymorphisms (SNP) based on the allele frequency, and thus had little biological significance.

Our investigation of associations of the *p53* mutational spectrum with mutations of oncogenic driver genes in OSCC did not produce any significant results. Chaudhary performed a comparison of the mutation rate of driver genes in head and neck SCC between African-Americans and Caucasians, and found high frequencies of *p53 *and *HRAS *mutations^39^. In our results, the mutation rates of *p53* and *NOTCH1* were high, but that for *PIK3CA* was low. These results might reflect the characteristics of the driver genes in Asians, but there are no previous data for comparison. There were 7 cases of wild-type *p53* (*p16-*negative) and an activating mutation of *HRAS* was found in 2 of these cases (codon 13 G13R and G13V). Mutation of *HRAS* was noted in only one (codon 12 G12S) of 31 cases with missense mutations, and there was no *HRAS* mutation in cases with truncation mutations. Mutation of *NOTCH1* was more frequent in wild-type *p53* cases than in mutant *p53* cases (including missense and truncation mutations). Although there was no significant association of mutations of *HRAS* and *NOTCH1* with the *p53* mutational spectrum, these findings may indicate that mutation of other major signaling pathways occurs at a high frequency in cancer cells with normal *p53* function.

Cases with *p53* mutation had significantly higher rates of pathological lymph node metastasis-positive status and Y-K mode of invasion ≥ 3, compared to wild-type cases. OS and DFS were significantly shorter in cases with lymph node metastasis, and DFS was significantly worsened by greater Y-K mode of invasion. Cases with *p53* truncation mutations also had a significantly shorter DFS. Use of the Kaplan–Meier method with a log-rank test showed no significant differences in OS or DFS among the 4 types in the *p53* mutational spectrum. However, a truncation mutation was a significant poor prognostic factor for DFS, but a missense mutation was not found as a prognostic factor for OS or DFS. Singh et al. reported *p53* mutational spectrum and its role in prognosis of oral cancer patients from India^40^. They mentioned that OS and DFS of OSCC patients with *p53* truncation mutation and transcriptionally non-active mutations were significantly lower than those with wild-type *p53*. These results were consistent with the results from our Japanese data. They examined 46 patients with OSCC and most of the patients (86.9%) were tobacco user (smoking and chewing). Although the tobacco habit in India was quite different from that in Japan, tobacco smoking and alcohol drinking was not related with the *p53* mutational spectrum in our study.

These findings suggest that *p53* function was completely lost in cases with truncation mutations, which increased malignancy. This may explain the significant differences between truncation and non-truncation mutation cases. In contrast, significant differences were not found between cases with *p53* missense mutations and other mutations in this study. This may have been due to the *p53* missense mutation cases being a mixture of those with mostly similar function to that of the wild-type, cases which loss of function, and cases with various degrees of gain of oncogenic function. We are planning to investigate possible acquiring functions based on the missense mutation site and pattern; i.e., a ‘oncogenic mutation of *p53*’, by preparing a database and identifying the biological characteristics and clinical behavior for each mutation. The current study might be positioned as the first step in preparation of ‘the mutational spectrum diagnostic database’.

There are about 200 amino acids in the DBD and about 20 kinds of amino acids can be substituted by mutation. Therefore, there are about 4,000 patterns of single amino acid substitutions. There were few cases with multiple mutations in the DBD in our study or in previous reports. These findings suggested that a single amino acid substitution was enough to change p53 protein function and no subsequent changes were required. Therefore, to prepare a database of functional changes corresponding to a mutation in the DBD, a search for a maximum of 4,000 mutants is sufficient. When we start a search for the clinical outcomes (invasive and metastatic potentials, and prognosis) as a result of functional abnormality (loss or gain of function) of *p53* in each case with known *p53* mutational site, it may enable realistic data accumulation. Separately, a search for functional analysis mutated *p53* products in vitro may help the construction of the database. We are planning to conduct the experiment and reported the preliminary results, in which transcriptional activity of mutated *p53* products for known target genes^26,27^ or for new target genes, or the ability to bind to *p63* and *p73* as a dominant negative effect are examined. By completing these databases, genome diagnosis based on the *p53* mutational spectrum may become possible and will provide important information for decision-making with regard to the treatment strategy for OSCC.

Neskey et al. proposed an evolutionary action score for p53 protein to stratify the tumors harboring *p53* mutation as high or low risk by computational approach^41^. They also validated this system in in vitro and in vivo. Patients with high risk *p53* mutations had poorest survival outcomes and shortest time to the development of distant metastasis. Tumor cells expressing high risk *p53* mutations were more invasive and tumorigenic and they exhibited a higher incidence of lung metastasis. Although our approach was somewhat different from their approach, the concept and the goal might be similar to those in their approach. Furthermore, Phase II clinical trial (ECOG-ACRIN 3132) in head and neck cancer harboring several patterns of *p53* gene (disruptive or non-disruptive *p53* mutation, *p53* wild-type) are ongoing^42^. We are also planning to conduct a clinical trial to select a treatment intensity (post-surgical chemo-radiation therapy and/or molecular targeted therapy) based on the *p53* mutational spectrum database.

## Data Availability

The data that support the findings of our study are available from the corresponding author upon reasonable request. Nucleotide sequence data reported are available in the DDBJ Sequenced Read Archive under the accession numbers DRA014726. https://ddbj.nig.ac.jp/resource/sra-submission/DRA014726.
